# Harnessing Natural Killer Cells in Non-Small Cell Lung Cancer

**DOI:** 10.3390/cells11040605

**Published:** 2022-02-10

**Authors:** Éilis Russell, Melissa J. Conroy, Martin P. Barr

**Affiliations:** 1 Cancer Immunology Research Group, Department of Physiology, School of Medicine, Trinity College Dublin, D02 R590 Dublin, Ireland; Russelei@tcd.ie (É.R.); Meconroy@tcd.ie (M.J.C.); 2 Thoracic Oncology Research Group, Trinity St. James’s Cancer Institute, St James’s Hospital, D08 W9RT Dublin, Ireland; 3 School of Medicine, Trinity Translational Medicine Institute, Trinity College Dublin, D08 W9RT Dublin, Ireland

**Keywords:** NSCLC, NK cells, NK cell therapies, immunotherapy, cancer

## Abstract

Lung cancer is the leading cause of cancer-related deaths worldwide. There are two main subtypes: small cell lung cancer (SCLC), and non-small cell lung cancer (NSCLC). NSCLC accounts for 85% of lung cancer diagnoses. Early lung cancer very often has no specific symptoms, and many patients present with late stage disease. Despite the various treatments currently available, many patients experience tumor relapse or develop therapeutic resistance, highlighting the need for more effective therapies. The development of immunotherapies has revolutionized the cancer treatment landscape by enhancing the body’s own immune system to fight cancer. Natural killer (NK) cells are crucial anti-tumor immune cells, and their exclusion from the tumor microenvironment is associated with poorer survival. It is well established that NK cell frequencies and functions are impaired in NSCLC; thus, placing NK cell-based immunotherapies as a desirable therapeutic concept for this malignancy. Immunotherapies such as checkpoint inhibitors are transforming outcomes for NSCLC. This review explores the current treatment landscape for NSCLC, the role of NK cells and their dysfunction in the cancer setting, the advancement of NK cell therapies, and their future utility in NSCLC.

## 1. Introduction

Cancer is a leading cause of death worldwide, with over 10 million deaths reported in 2020 alone. Lung cancer was only recently surpassed as the most common cancer type, yet it remains the primary cause of cancer-related mortality, with almost 1.8 million individuals succumbing to the disease [1]. Lung cancer is classified as either small cell (SCLC) or non-small cell lung cancer (NSCLC). NSCLC accounts for approximately 85% of all lung cancer diagnoses and can be further subdivided into several subtypes: adenocarcinoma (ADC), squamous cell carcinoma (SqCC), large cell carcinoma (LCC), and mixed histology [2]. ADC arises from alveolar cells and represents approximately 40% of NSCLC cases. Conversely, SqCC arises from the bronchial epithelium and accounts for 25–30% of diagnoses [3]. The average 5-year survival rate for NSCLC varies and is dependent on cancer stage; rates range, from 63% for Stage I NSCLC, to a dismal 7% for Stage IV NSCLC [4]. While early stage lung cancer, for most patients, is resectable with curative intent, approximately 70% of patients present with advanced stage disease [5]. This has highlighted the need for early diagnosis and more effective therapies, so as to improve survival outcomes for patients.

## 2. Clinical Challenges Associated with Non-Small Cell Lung Cancer

Cigarette smoking, current or former, is the most common risk factor associated with the development of lung cancer and is responsible for approximately 80% of cases [6]. SqCC is more commonly associated with smoking and chronic inflammation in contrast to ADC, which is becoming increasingly diagnosed in never-smokers and tends to occur more often in younger females [7]. Smokers tend to have higher mutation frequencies in Kirsten rat sarcoma (KRAS) and tumor protein P53 (TP53), whereas higher levels of actionable mutations, such as epidermal growth factor receptor (EGFR), ros-oncogene 1 (ROS1), and anaplastic lymphoma kinase (ALK), are associated with never-smokers [8]. Other risk factors include the use of other tobacco products, exposure to second-hand smoke, occupational carcinogens, radiation, radon, and air pollution [6].

In terms of treatment modalities, surgical resection with curative intent is the most effective treatment for stage I-IIIA NSCLC [3]. Patients can also be offered a combination of radiation therapy, chemotherapy, and/or surgery, depending on variables such as tumor size, location, lymph node involvement, underlying co-morbidities, and tolerance to treatment [9]. Immunotherapies can also be administered, as either first-line treatment or as an adjuvant therapy [9]. In advanced disease, where targeted or immunotherapies are not possible, platinum-based chemotherapy such as cisplatin is often used [10]. However, cisplatin resistance has become a significant clinical challenge in these patient cohorts.

NSCLC is a molecularly heterogeneous disease, comprising various genetic abnormalities that further subdivide the disease into specific molecular subtypes. EGFR mutations are one of the most common alterations observed in NSCLC and occur most frequently as either exon 19 deletions or exon 21 L858R mutations [11]. FDA-approved targeted treatments for EGFR mutations include the tyrosine kinase inhibitors erlotinib, gefitinib, afatinib, osimertinib, and dacomitinib. In advanced stage disease, most of these therapies are administered as monotherapies. However, erlotinib can be used in combination with angiogenesis inhibitors such as bevacizumab and ramucircumab in advanced stage NSCLC, while osimertinib can be used as an adjuvant treatment post-surgery for early-stage NSCLC [12,13]. These therapies are not as effective in patients with EGFR exon 20 insertion mutations; however, the FDA has recently approved therapies for targeting this mutation, including mobocertinib and amivantamab, which are typically administered after failure of prior platinum based-chemotherapy [12,13].

Mutations in KRAS are also frequently observed in NSCLC. Approximately 13% of patients present with a specific mutation, KRAS G12C. This mutation is often resistant to other targeted therapies such as EGFR inhibitors [13]. Sotorasib received FDA-approval in June 2021 as a first-line treatment for individuals with this genetic mutation after treatment of at least one prior systemic therapy [12]. These aforementioned genetic mutations are most frequently observed in ADC alongside mutations in tumor-suppressor genes *TP53*, Kelch-like ECH-associated protein 1 (KEAP1), serine/threonine kinase 11 (STK11), and neurofibromin 1 (NF1) [8]. Less common genetic mutations include mutations in ALK, ROS1, B-Raf proto-oncogene serine/threonine kinase (BRAF), rearranged during transfection proto-oncogene (RET), and mesenchymal epithelial transition (MET). Approximately 5% of NSCLC diagnoses are EML4-ALK-positive, where this fusion protein is commonly observed in young patients and non-smokers [13]. Loratinib is the first-line treatment for this subtype, but additional therapies including crizotinib, certinib, alectinib, and brigatinib are also used [12,13]. Mutations in ROS1 are rare, but the gene rearrangement shares similarities with ALK gene rearrangements. Therapies that can be administered for ALK mutations include crizotinib, certinib, and lorlatinib, which act by inhibiting ALK enzyme activity [14]. Entrectinib can be administered for individuals with metastatic NSCLC with ALK mutations. Therapies targeting BRAF mutations include dabrafenib and trametinib, which can be used in combination for metastatic NSCLC. RET inhibitors such as selpercatinib and pralsetinib target the abnormal RET protein, while MET inhibitors such as capmatinib and tepotinib can be used to treat metastatic NSCLC characterized by MET gene mutations [13]. However, these therapies continue to present a significant clinical challenge, in that they are only effective in individuals harboring actionable mutations, after which time, drug resistance eventually develops. As observed with targeted therapies, treatment options for NSCLC have evolved from a ‘one size fits all’ approach to a more personalized strategy based on the specific tumor characteristics.

The introduction of immunotherapies has revolutionized the cancer landscape, improving response and survival rates for patients with advanced stage NSCLC [15]. Current immune checkpoint inhibitors (ICIs) have the ability to target several immune checkpoint signals, including the classical programmed cell death protein 1 (PD-1), programmed cell death ligand 1 (PD-L1), and cytotoxic T lymphocyte-associated protein (CTLA-4) checkpoints, in addition to new emerging checkpoints such as T cell immunoglobulin and mucin domain-containing protein 3 (TIM-3), lymphocyte activation gene-3 (LAG-3) and T cell immunoreceptor with Ig and ITIM domains (TIGIT) [15]. Anti-PD-1/PD-L1 therapies have demonstrated efficacy in many malignancies in NSCLC. Anti-PD-1 monoclonal antibodies (mAbs) such as nivolumab, pembrolizumab and cemiplimab, and anti-PD-L1 mAbs atezolizumab and durvalumab target PD-1/PD-L1 binding function, by essentially removing the brakes from the anti-tumor immune response [13]. These immunotherapies are offered as a first-line therapy for NSCLC or in conjunction with other immunotherapies or chemotherapy [13]. Despite their effectiveness, ICIs are not a panacea and do not fully address the treatment requirements of all NSCLC patients. It has been shown that up to 30% of patients show rapid progression, while only 10–15% of patients demonstrate long term benefits [16]. In addition, the use of ICIs leads to immunotherapy-induced resistance, presenting another significant clinical challenge in NSCLC [17]. The combination of multiple ICIs has also demonstrated improved clinical responses. In a review by Ma et al., the presence of other untargeted immune checkpoints was highlighted, possibly compensating for the blockade of specific signals [15]. This provides a rationale for combining ICIs, and many trials have investigated the utility of this combinatorial approach and have found its superiority over monotherapy [18,19].

Despite recent advances in treatment options, survival rates for NSCLC patients with advanced disease remain poor. Many challenges are yet to be overcome, as detailed by Ahluwalia et al. [20]. These include tumor heterogeneity, drug resistance, and a lack of reliable biomarkers that can reliably stratify patients into those that may be poor or good responders. Further work to elucidate such factors pertaining to these challenges will aid in the advancement of current and novel treatment modalities.

## 3. Natural Killer Cells in Health and Disease

Natural killer (NK) cells were first described in the 1970s and belong to a subgroup of the innate lymphoid cell family [21]. They arise from common lymphoid progenitors, but unlike T cells and B cells, they lack genetically rearranged receptors, are independent of antigen specificity and rely on a balance of signals transduced via activating and inhibitory receptors to induce activation [22]. NK cells represent 5–15% of circulating lymphocytes and are present primarily in the bloodstream and in lymphatic vessels [23,24]. They also act as sentinels in various other sites, such as the liver, bone marrow, and the lungs [23]. The heterogenous NK cell population is commonly and broadly characterized based on their maturation status and expression levels of CD56 and CD16 [23,25]. Approximately 90% of all peripheral blood NK cells are classified as CD56^dim^CD16^bright^. This subset of NK cells are highly cytotoxic, produce modest levels of cytokines, and express markers such as CD16, CD57, and PEN5. By contrast, the CD56^bright^CD16^-^ subset mainly reside in secondary lymphoid organs and express markers such as CD122, NKp46, and NKp80. These CD56^bright^ NK cells produce a larger number of cytokines and chemokines compared to the CD56^dim^ subset and are less cytotoxic [26,27,28]. It has been shown that following activation, the immature CD56^bright^ NK cells increase their expression of NK receptors characteristic of the more mature and cytotoxic CD56^dim^ subset [29,30,31].

**Figure 1:**
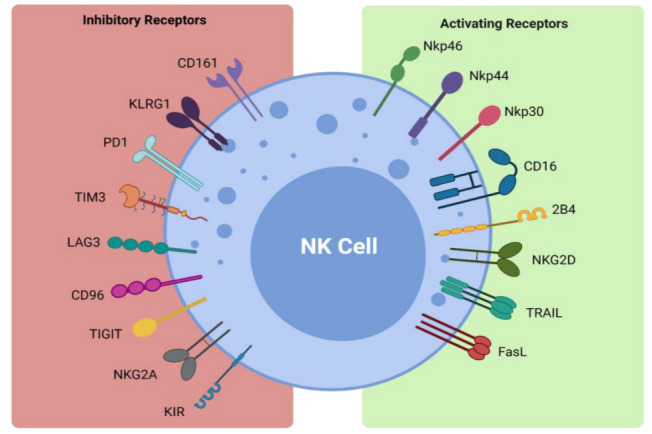
Activating and inhibitory NK cell receptors. Figure illustrating the panel of inhibitory and activating receptors expressed on the surface of NK cells that regulate their activation and effector function (www.biorender.com, accessed 21 December 2021).

NK cells form part of the first line of defense in mediating viral infections and cancer immune surveillance, particularly cancer metastases. They exercise their cytotoxic effects without the need for pre-activation via the release of perforin and granzymes [32]. The cytotoxic functions of NK cells are induced by a delicate interplay between activating and inhibitory NK cell receptors (Figure 1) and their cell-surface bound ligands (Table 1). Inhibitory receptors such as killer cell immunoglobulin-like receptors (KIRs) and the CD94-NKG2A complex have the ability to recognize self-molecules on the surface of normal cells and inhibit NK cell activation [26,33]. In addition, the immunoreceptor tyrosine-based inhibitory motifs (ITIMs) of certain inhibitory receptors can interact with MHC class I molecules to aid in the processes of NK cell licensing and education, ensuring full activation and self-tolerance of NK cells [26].

Activating NK cell receptors include receptors such as NKG2D and the natural cytotoxicity receptors NKp30, NKp44, and NKp46 [26,40,41,42,43]. They bind to stress-induced self-ligands on infected or malignant cells and trigger NK cell cytokine production and cytotoxicity, while killer activating receptors upregulate death ligands such as TNF-α, FasL, and TRAIL, which are key components in the apoptosis of target cells [32,37,38]. The CD16 receptor is another activating receptor that is responsible for the triggering of antibody-dependent-cellular cytotoxicity (ADCC) against antibody- coated target cells [26]. Infected or transformed cells often downregulate their MHC class I expression to evade detection by T cells and their absence can activate NK cells in a process coined ‘missing-self recognition’ [26]. NK cells can also modulate the immune response via interaction with other immune cells, such as cross-presentation of antigens from apoptotic target cells to particular subsets of dendritic cells and priming of CD4+ helper T cells via interferon-γ (IFN-γ) production, as reviewed by Vivier et al. [26,44,45]. Importantly, NK cells can also promote resolution of an immune response by killing activated T cells or via suppression of autoreactive B lymphocytes in vitro [26,46,47]. Therefore, NK cells are not only potent killers of transformed cells but also promote tumor eradication via their recruitment and activation of other arms of the immune response.

## 4. NK Cells in NSCLC

The majority of the NK cell population in the lung is CD56^bright^CD16^-^, exhibiting high cytokine release but low cytotoxicity [48]. In fact, in vivo studies have demonstrated that human lung NK cells respond poorly to activation by target cells, when compared to peripheral blood NK cells. It has been proposed that this is due to the suppressive effects associated with alveolar macrophages and soluble factors that are present in the epithelial lining of the lower respiratory tract to maintain lung homeostasis [48,49]. These human lung NK cells share several phenotypic similarities with the decidual NK (dNK) cells present in the maternal decidua during pregnancy [50]. Such dNK cells are involved in promoting invasion of the invasive extravillous trophoblasts, vascular remodeling, and the establishment of fetal tolerance during pregnancy via the production of various cytokines/chemokines and pro-angiogenic factors [50]. These dNK cells have been shown to produce several pro-angiogenic factors such as vascular endothelial growth factor (VEGF), placental growth factor (PlGF), and NKG5 and are potent secretors of IL-8 [51]. The CD56^bright^CD16^dim^ subset that are enriched in NSCLC tumor samples have shown similarities to dNK cells, in that they produce some of the same pro-angiogenic factors, such as VEGF, PlGF, and IL-8, promoting tumor growth and metastases [52]. While this NK cell phenotype in the maternal decidua permits the establishment and maintenance of pregnancy and is beneficial, this NK cell subtype in NSCLC is more likely to be harmful through the promotion of tumorigenesis and certainly warrants further investigation. Multiple studies have reported that the infiltration of NK cells into solid tumors is associated with favorable prognosis in many cancers [53,54]. In lung cancer, downregulated NK cell receptor expression has been reported on intratumoral NK cells, while defective degranulation and IFN-γ production has also been observed [55]. Therefore, the restoration of NK cell responses using NK cell therapies is a desirable therapeutic concept in this malignancy.

### Suppression and Evasion of NK Cell Responses within the NSCLC Tumor Microenvironment

**Figure 2:**
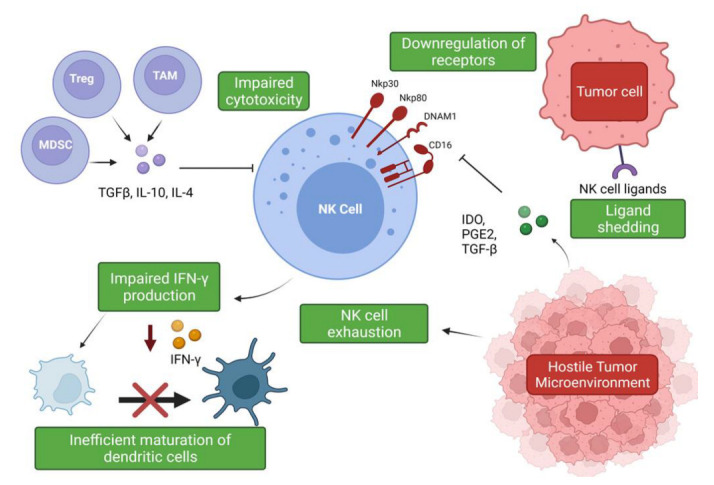
Suppression and evasion of NK cells in the tumor microenvironment. Figure illustrating the various factors that contribute to NK cell dysfunction in the TME and the impact on other immune cells (www.biorender.com, accessed 27 December 2021).

The hostile tumor microenvironment (TME) can impair NK cell functions by attenuating their cytotoxic capabilities via the production of immunosuppressive factors and via nutrient deprived and hypoxic conditions [56] (Figure 2). Furthermore, the TME’s role in propagating pro-tumor responses presents a significant clinical challenge when designing therapeutics.

The hypoxic conditions of the TME are responsible for the promotion of angiogenesis via induction of hypoxia-inducible factor-1 (HIF-1), leading to the production of pro-angiogenic factors such as VEGF [57,58]. Experimental evidence has shown that a hypoxic environment combined with TGF-β1 and Aza, a demethylating agent, enriched peripheral NK cell cultures in CD56^bright^CD16^dim^ cells and induced secretion of VEGF, leading to increased angiogenesis [59]. Phenotypic conversion of this subset to pro-angiogenic, decidual-like NK cells has been observed in NSCLC tumor samples, with an increased production of VEGF, PlGF, and IL-8 observed in patients with both ADC and SqCC [52]. Exposure to TGF-β1 upregulates VEGF and P1GF production in the NK cells of healthy controls, suggesting that the pro-angiogenic phenotype associated with NSCLC is, in part, mediated by TGF-β1 [52]. Another member of the innate lymphoid group, type 2 innate lymphoid cells (ILC2s), has also been implicated in promoting angiogenesis and cancer progression. ILC2s have emerged as a major driver of type 2 inflammation, producing typical type 2 cytokines such as IL-4, IL-5, and IL-13 and contributing to the pathogenesis of inflammatory conditions such as asthma [60]. In the context of NSCLC, ILC2s have been shown to be enriched in NSCLC tumor samples and to upregulate PD-1 expression, displaying an increased expression of type 2 cytokines such as IL-4 and IL-13, suggesting these immune cells also contribute to the immunosuppressive environment [61]. IL-33 production driven by these cytokines is thought to be responsible for the pro-tumor activity of these immune cells, leading to increased angiogenesis and tumor metastases [62,63].

Soluble factors expressed by lung cancer cells have been associated with a decreased expression of granzyme B, perforin, and IFN-γ by infiltrating immune cells, including NK cells [64]. Tumor-associated macrophages (TAMs) and myeloid-derived suppressor cells (MDSCs) produce immunosuppressive factors such as TGF-β, IL-10, and PGE2, which impede NK cell cytotoxicity [27]. The secretion of IL-10 by TAMs suppresses NK cell cytotoxicity, and an increase in IL-10 production alongside reduced IFN-γ production has been described in patients with NSCLC [27,65]. IL-10 overproduction has been associated with enhanced angiogenesis, and, moreover, an increase in serum IL-10 levels is an indicator of poor prognosis [65,66,67]. However, IL-10 can significantly increase glycolysis and enhance the effector functions of NK cells via mTORC1 signaling [68]. These findings suggest a potential use of this classical immunomodulatory cytokine in the promotion of NK cell effector functions and warrants further investigation. TGF-β regulates NK cell function by inhibiting the expression of NKp30 and NKG2D receptors, which are vital in the recognition and destruction of tumor cells [27,69]. This inhibition has been suggested to negatively impact NK-cell mediated killing of immature dendritic cells (DC), which is an important mechanism for eliminating potentially tolerogenic DCs, in controlling the adaptive immune response [69,70]. The bi-directional cross-talk between NK cells and DCs is important for their activation and maturation, respectively [71]. IFN-γ production has been shown to be vital for the activation of endogenous DCs [71,72], but it has been shown that intra-tumoral NK cells are impaired in their ability to secrete IFN-γ; therefore, negatively impacting DC maturation [27,73]. It has also been shown that TGF-β drives NK cell dysfunction in the lung tumor microenvironment via FBP1 upregulation and subsequent inhibition of glycolysis in lung NK cells [74].

The rate-limiting enzyme involved in tryptophan catabolism, indoleamine 2,3-dioxygenase (IDO), is released by tumor cells and can inhibit the upregulation of NKp46 and NKG2D via blockade of IL-2, again limiting the NK cell’s ability to recognize and kill tumor cells [75]. Cigarette smoking has also been implicated in NK cell dysfunction and has been shown to reduce their cytotoxic abilities. Smokers have been shown to exhibit a significant reduction in the secretion of IFN-γ and TNF-α when compared to non-smokers. Interestingly, it was found that this inhibitory effect on TNF-α production was reversible, suggesting that smoking cessation may somewhat improve NK cell responses to cancer [76]. IL-15 plays an important role in the proliferation, survival, and function of NK cells, and its production was also found to be suppressed due to the effects of cigarette smoking; thereby, negatively impacting critical mechanisms of NK cell development and function [77]. These findings stress the need to promote smoking cessation, as it not only drives cancer development but is also a promotor of progression via impairment of the immune response. As NK cell dysfunction and impairment promotes tumor immune evasion in NSCLC, emerging therapies have focused on restoring normal function of NK cells by means of cytokine supplementation, blockade of inhibitory receptors, and neutralization of immunosuppressive cytokines such as TGF-β [73].

NK cells have also been shown to become exhausted as a result of the hostile TME, leading to impaired effector function and an altered phenotype [78]. NK cell exhaustion has been shown to be responsible for the significant decrease in NK cell cytotoxicity in lung cancer patients [79]. Moreover, the NKG2A receptor has been shown to be upregulated, whereas the CD226 receptor has been found to be downregulated in these patients [79]. The expression levels of these receptors may prove useful as markers of NK cell exhaustion and as potential therapeutic targets in this cohort.

Another common evasion strategy employed by tumors to avoid NK cell-mediated immune detection is NK receptor ligand shedding. NKG2D ligands are normally absent or expressed at low levels on healthy cells but become upregulated on tumor cells, allowing for detection by immune cells [80]. However, tumor cells can shed their ligands via proteases such as matrix metalloproteinases (MMPs) and members of a disintegrin and metalloprotease family (ADAMs), which allows them to avoid immune detection. Furthermore, the presence of soluble NKR ligands in patient sera is often correlated with poor prognosis, and it has been suggested that the hypoxic conditions of the TME may promote the accumulation of such soluble ligands [81,82,83]. Moreover, it has been shown that soluble NKG2D ligands contribute to cell senescence and are a part of the senescent secretome [81,84,85]. Senescence is another tumor-suppressive mechanism that has been shown to contribute to tumor relapse and the adverse effects associated with chemotherapy [81]. The NKG2D ligand recognizes MHC class I chain-related protein A and B (MICA/B) alongside UL16-binding proteins (ULBPs), and roles for ADAM9, ADAM10, and ADAM17 have been elucidated in the shedding of these molecules. ADAM10, specifically, has been implicated in the immune evasion strategies employed by NK cells and is a potential therapeutic target to prevent the shedding of NKG2D ligands [81]. The many immunosuppression and evasion strategies of tumor cells illustrate the complexity of the disease and stress the need for a better understanding of the impact of the TME on NK cell function, to develop effective NK cell-based therapeutics.

## 5. NK Cell Therapies for NSCLC

The critical role of NK cells in cancer has been well established, and many studies are exploring their potential as the basis for novel cancer therapeutics. The efficacy of NK cell therapies is currently being explored and trialed in various cancer types. Examples of NK cell therapies include administration of autologous and allogeneic NK cells, CAR-NK cells, cytokine supplementation, and monoclonal antibodies (Table 2).

### 5.1. Adoptive Transfer of NK Cell Therapies

As previously discussed, expanding NK cell populations ex vivo, is a desirable therapeutic concept to enhance NK cell tumor responses. Autologous NK cell populations can be obtained from the patient’s own blood, or in the case of allogeneic populations, from either umbilical cord blood, cell lines, or from other adult healthy donors. However, autologous NK cells obtained from pre-treated patients have been shown to be impaired in their expansion efficiency and functional status compared to allogeneic NK cells [22,86]. Previous attempts to improve expansion rates via administration of high dose IL-2 in vivo only led to significant toxicity, as reviewed by Geller et al. [87]. It was thought that the failure of autologous NK cell therapies could be partially attributed to inhibitory KIRs inability to recognize self-MHC class I molecules on the surface of tumor cells. Following on from this, research began to focus on using allogeneic NK cells. Allogeneic NK cells derived from adult healthy donor cells are advantageous, in that they have already been educated in healthy hosts and, thus, have greater potential in antitumor activity [87]. Their use has been successful in the treatment of hematological malignancies such as in acute myeloid leukemia (AML) [88], and research has expanded to investigate their utility in solid tumors. Their use in NSCLC holds promise, with a recent study by Lin et al. demonstrating their efficacy in combination with pembrolizumab in improving survival for advanced stage patients [89]. However, as noted by Geller et al., limitations still remain with these allogeneic NK cells; in particular, the possibility of the failure of donor NK cells to expand in vivo. Thus, the success of NK cell expansion remains unpredictable in patients with solid tumors [87]. This further illustrates the need for prior profiling of the TME and its immunosuppressive factors before the adoptive transfer of NK cells, as well as modification of NK cell therapies to arm them for a hostile microenvironment.

The adoptive transfer of NK92 cell lines has been proven safe in humans after multiple infusions [90]. These cell lines can be easily genetically modified to improve efficacy and overcome immune suppression in the TME. For example, NK92 can be modified to express the high-affinity CD16 FcγRIIIa receptor, to facilitate antibody-dependent cell cytotoxicity [91]. These high-affinity NK (haNK) cells also endogenously express IL-2; therefore, mitigating the need for culture with exogenous IL-2. The haNK cells have been shown to efficiently lyse human H441 lung carcinoma cells and have progressed to clinical trials for multiple solid malignancies (NCT03586869, NCT03387085, NCT04927884, NCT04390399, NCT04847466), including NSCLC (NCT03228667).

Modification of NK cells to improve their efficacy is a promising area of growth in the NK cell therapy space. Following the success of chimeric antigen receptor (CAR)-T cell therapies for hematological malignancies, CAR-NK cells hold significant promise as an ‘off-the-shelf’ alternative. CAR-NK cells hold advantages over CAR-T cell therapy, as they are MHC-independent, do not induce graft-versus-host disease, and have a limited lifespan, foregoing the need for insertion of suicide genes [92]. The majority of clinical trials are investigating the CAR-NK cells using the NK92 cell line [92]. CAR-NK cells directed at the NK cell receptor ligand B7-H3 have demonstrated efficacy in limiting tumor growth in mouse xenografts of NSCLC, providing a rationale for their use in cancer immunotherapy [93]. A clinical trial investigating the use of anti-54T CAR-NK cell therapy in locally advanced or metastatic solid tumors, including NSCLC, is currently recruiting (NCT05137275). The versatility of designing CAR-NK cells to target multiple cell surface molecules provides promise in the treatment of NSCLC.

### 5.2. NK Cell Cytokine Therapies

Cytokine supplementation is another modality whereby NK cell function can be enhanced. IL-2 is known to promote the proliferation, cytotoxicity, and cytokine secretion of NK cells. However, it also expands T-regulatory (Treg) cell populations, limiting the anti-tumor functions of NK cells. Variants of IL-2 such as NKTR-214 have been developed that only promote a minor expansion of Tregs, while enhancing anti-tumor responses [22]. Clinical trials are currently underway investigating the use of NTKR-214 treatment in NSCLC exclusively, in addition to other advanced or metastatic solid tumors (NCT03138889, NCT03548467, NCT02983045). IL-15-based therapy is superior to IL-2 therapy, in that it does not expand Treg populations. However, it does play a role in NK cell exhaustion and, therefore, it has been proposed as an intermittent therapy [22]. The use of the IL-15 superagonist, ALT-803, in combination with nivolumab has shown promise in a Phase Ib trial [94]. Phase II of this trial is currently underway with a larger cohort of participants and will provide greater insights into its efficacy across several NSCLC subtypes (NCT02523469). Cytokine therapies are providing promising effects due to their potential to activate and expand a patient’s own NK cells. Whilst there are drawbacks to their use, due to their potential to evoke off-target immune responses, exploring their administration with other therapies such as ICIs and haNK cell therapies is currently under investigation in Phase I, Ib, and II clinical trials across multiple malignancies, including NSCLC (NCT03138889, NCT02983045, NCT02523469).

### 5.3. Monoclonal Antibodies

Monoclonal antibodies (mAbs) have been developed to target various NK cell receptors. Monalizumab targets the NKG2A receptor on both T cells and NK cells [22]. Its use is currently being investigated in combination with durvalumab in a Phase II trial in subjects with locally advanced, unresectable, stage III NSCLC (NCT03822351). This combination is also being trialed in a Phase II trial for patients with PD-1 ICI-resistant advanced NSCLC (NCT03833440). The combination was also explored in a Phase II trial for early stage, resectable NSCLC (NCT03794544). Lirilumab is a mAb that can target the inhibitory KIR receptors present on the NK cell, and its efficacy has been documented in preclinical investigations of multiple myeloma (MM) and AML [22,95,96]. Its use was explored in combination with epacadostat, an IDO inhibitor, and nivolumab in advanced/metastatic malignancies including NSCLC (NCT03347123). Another mAb, elotuzamab, targets the SLAM7 receptor and pre-clinical mouse models of MM provide a rationale for trialing its use in combination with anti-PD1 treatment, to promote the infiltration of NK cells and increase intra-tumoral cytokine and chemokine release [97]. TIGIT is a novel checkpoint target, and antibodies targeting this checkpoint have proven effective. A novel anti-TIGIT mAb, AET2010, holds promise for preventing NK cell exhaustion and inducing anti-tumor NK cell immunity, both ex vivo and in vivo [98].

Bispecific killer cell engagers (BiKEs) are bispecific mAbs that can target both NK cell receptors and tumor antigens. They have the ability to trigger NK cell-mediated lysis of tumor cells in hematological malignancies [22] and are currently under investigation in solid malignancies. A study conducted by AstraZeneca to assess the safety and efficacy of an anti-TIGIT/anti-PD-1 bispecific antibody, AZD2936, in NSCLC is currently in a Phase II trial (NCT04995523). HLX301, an anti-TIGIT/anti-PD-L1 bispecific antibody is also being investigated in patients with NSCLC; however, the study is not yet recruiting (NCT05102214). With promising results in preclinical MM studies and with the various clinical trials currently underway, these mAbs are a promising avenue to explore for the treatment of NSCLC. The use of AFM24, a tetravalent, bispecific EGFR/CD16A innate cell engager, is under investigation in various trials. A trial investigating its utility as a monotherapy is currently recruiting and will include multiple cancer types, such as renal cell carcinoma (clear cell), NSCLC with EGFR mutations, and colorectal cancer (NCT04259450). Its use will also be trialed in combination with the PD-L1 checkpoint inhibitor atezolizumab in EGFR-expressing solid tumors for NSCLC (NCT05109442). Another combinatorial trial of AFM24 and the autologous NK cell therapy SNK01 is currently recruiting patients for NSCLC (NCT05099549).

## 6. The Future of NK Cell Therapies in NSCLC

The various treatment modalities currently in trial for NSCLC hold promise for the future development of novel therapeutics to tackle the existing clinical challenges associated with NSCLC. Some additional novel strategies are also being explored, including the targeting of autophagy. It has been shown that cancer cells can resist NK cell-mediated killing by decreasing granzyme B levels via autophagy, more specifically via activation of ULK1. A study by Yao et al. demonstrated that by inhibiting autophagy using rocaglamide, it was possible to block autophagic immune resistance to NK cell-mediated killing in NSCLC, by repressing ULK1 protein translation [99]. This repression led to improved NK cell-mediated cell lysis and is a promising future target for NSCLC treatment.

Following on from the success of BiKEs, trispecific killer cell engagers (TriKEs) have been developed that contain two antibody fragments against CD16 and CD33, alongside an immune stimulatory cytokine crosslinker such as IL-15 [100]. The 161533 TriKE was shown to induce profound NK cell cytotoxicity, degranulation, and cytokine production in the AML HL-60 cell line [100]. Despite their effectiveness in preclinical tumor models of hematological malignancies, no clinical trials are currently investigating their utility in solid tumors, but they may have potential for further study. Future work could explore designing these killer cell engagers to target multiple NK cell receptors in NSCLC and perhaps cross-linking other cytokines that are known to potentiate NK cell expansion and function, such as IL-2 and IL-18.

NK cell immunotherapy is a promising therapeutic strategy; however, it is not without its limitations. As reviewed by Kim et al., several challenges remain regarding the efficacy of NK cell therapies, including increasing the activity, infiltration, and homing of NK cells, alongside promoting contact between NK cells and tumor cells [101]. The emergence of nanomedicine is an exciting and promising approach to resolving these aforementioned limitations, by aiding in the delivery and efficacy of NK cell therapies. Nanotechnology can also provide alternative routes of administration, for example, via inhalation, which is particularly relevant to NSCLC. This mode of administration allows for direct entry into the lungs, bypassing first-pass metabolism and reducing systemic toxicity [102]. Tumor suppressor candidate 2 (TUSC2) nanovesicle-based immunogene therapies, when combined with anti-PD-1 therapy, were shown to significantly inhibit tumor growth and extend survival in mouse models of Kras-mutant lung cancer [103]. The success of this nanomedicine in combination with ICIs warrants further trials as a potential therapy. The use of nanoparticles has also been shown to aid in reducing the adverse systemic toxicity observed following administration of immunostimulatory agents [104]. Anchoring of anti-CD137 and IL-2 to PEGylated liposomes allowed for an effective anti-tumor response, without adverse systemic effects, in multiple tumor models [104]. Nanomedicine can also aid in promoting NK cell expansion, which is crucial to elicit a robust NK cell-mediated anti-tumor response. Ex vivo, PM21 particles were shown to enhance NK cell expansion from peripheral blood mononuclear cells (PBMCs) of both healthy donors and patients with AML and, moreover, were shown to stimulate in vivo NK cell expansion in mouse models, providing a promising method by which to promote sufficient NK cell expansion [105]. Nanomedicine is a promising therapeutic adjuvant to boost NK cell cytotoxicity and expansion, allowing for more robust anti-tumor responses.

Another potential therapy that may prove useful in NSCLC is administration of PARP inhibitors in combination with NK cell therapies. Poly-ADP ribosylation (PAR) is a post-translational modification that is catalyzed by poly-ADP ribose polymerase (PARP) and plays an important role in repairing single-stranded DNA breaks [106]. PARP inhibitors prevent the single-stranded break repair, which leads to the induction of double-strand breaks. Double-strand breaks are repaired by homologous recombination, which has been shown to be disrupted in 10–15% of NSCLC patients [106]. The PARP inhibitor, veliparib, has been trialed in combination with carboplatin/paclitaxel for patients with non-squamous NSCLC. Despite its tolerability in combination with chemotherapy, no significant benefit of treatment was found (NCT02264990). The PIPSeN study investigated the use of olaparib as a maintenance therapy but was terminated following the registration of anti-PD-L1 agents as a first-line treatment [107]. The trial reported that olaparib was not associated with an improved survival. However, since the study was statistically underpowered and the drug was well tolerated, further work is warranted on the utility of this drug. In pre-clinical studies, olaparib has been shown to upregulate NKG2D ligand expression on the surface of the AML cell line, HL-60, and promoted the cytotoxicity of the NK cells [108]. Furthermore, olaparib has been tested in combination with haNK cells and has been shown to increase TRAIL receptor expression and tumor sensitivity to NK cell-mediated cytotoxicity across multiple tumor types [109]. Olaparib has also been reported to significantly enhance NK cell-mediated killing of non-small cell lung carcinoma cells [109]. Therefore, there is potential to examine the feasibility of PARP inhibition in combination with NK cell therapies such as haNK cells in NSCLC.

## 7. Conclusions

Despite the availability of a variety of systemic anti-cancer treatments for NSCLC, the prognosis for many patients remains dismal. Immunotherapies have revolutionized cancer treatment for patients with NSCLC, but they are not without their limitations. Novel approaches to patient stratification, combination treatments, and treatment sequencing are needed. The pivotal role of NK cells in anti-cancer immunity warrants further investigation regarding their utility as potential therapeutic targets in the context of NSCLC. NK cell-based therapies are a promising and exciting avenue to explore as an ‘off-the-shelf’ approach to cellular therapy, particularly in combination with existing immunotherapies. Their genetic modification promises to help overcome the NK cell dysfunction and suppression observed in NSCLC patients. Furthermore, NK cells offer a superior alternative to T cells for cellular therapies, as they do not require antigen specificity, they do not need to be sourced from pre-treated patients, and facilitate easier scale-up and more efficient transfers to recipients. However, further studies are warranted to achieve better tumor infiltration, boost cytotoxicity, and prolong persistence. Overall, NK cell-based therapy is a promising treatment modality to add to the emerging development of therapeutics in NSCLC. Future evaluation of their efficacy in combination with other immunotherapies may unlock their potential for improving treatment response and survival outcomes in NSCLC patients.

## Figures and Tables

**Table 1:** NK cell receptors and ligands [26,34,35,36,37,38,39].

Receptor	Ligand
Inhibitory Receptors and Ligands	
CD161	LLT1
KLRG1	Cadherins
PD-1	PD-L1
TIM3	Galectin 9, phosphatidylserine, CEACAM1, HMGB1
LAG3	MHC class II
CD96	CD155
TIGIT	CD155, CD112, CD113
NKG2A	HLA-E
KIR	HLA-C/B/A
Activating Receptors and Ligands	
NKp46	Viral hemagglutinins
NKp44	Viral hemagglutinins
NKp30	PP65, BAT-3
CD16	IgG
2B4	CD48
NKG2D	ULBP, MICA/B
TRAIL	TRAIL-R1, TRAIL-R2
FasL	Fas

**Table 2:** NK cell-based immunotherapies in clinical trials for NSCLC.

Study	Modality	Phase	Intervention
NCT04628780	Cytokine	I	Anti-PD-1 targeting IL-15 fusion protein, PF-07209960
NCT03987867	Autologous NK cells	I	Autologous CIK cell immunotherapy in combination with PD-1 inhibitor and chemotherapy as a first line treatment
NCT05137275	CAR NK cells	Early Phase I	Anti-5T4 CAR NK cells
NCT03138889	Cytokine	I/II	NKTR-214 in combination with pembrolizumab with or without chemotherapy
NCT03548467	Cytokine	I/II	NKTR-214 in combination with VB10.NEO
NCT02983045	Cytokine	I/II	NKTR-214 in combination with nivolumab and/or ipilimumab
NCT02523469	Cytokine	I/II	ALT-803 in combination with nivolumab
NCT03347123	IDO inhibitor + immunotherapies	I/II	Epacadostat in combination with nivolumab and ipilimumab Epacadostat in combination with nivolumab and lirilumab
NCT04259450	BiKE	I/IIa	AFM24 monotherapy
NCT05109442	BiKE	I/IIa	AFM24 in combination with atezolizumab
NCT05099549	BiKE + autologous NK cell therapy	I/IIa	AFM24 in combination with SNK01
NCT04995523	mAb	I/II	Anti-TIGIT/anti-PD-1 bispecific antibody AZD29636
NCT05102214	mAb	I/II	Anti-TIGIT/anti-PD-L1 bispecific antibody HLX301
NCT03474497	Cytokine + ICI/radiotherapy	I/II	IL-2 in combination with pembrolizumab and radiotherapy
NCT04872634	Autologous NK cell therapy	I/IIa	SNK01 in combination with chemotherapy or chemotherapy/cetuximab
NCT04616209	Allogeneic NK cell therapy	I/II	PB103 allogeneic NK cells
NCT03822351	ICI	II	Durvalumab alone vs. durvalumab in combination with oleclumab/monalizumab
NCT03833440	ICI in combination with other immunotherapies/chemotherapy	II	Durvalumab + monalizumab Durvalumab + oleclumabDurvalumab + AD6738Docetaxel
NCT03794544	ICI	II	Durvalumab Durvalumab + oleclumab/monalizumab/danvatirsen
NCT03789604	mAb	III	CS1001 in combination with platinum-containing chemotherapy
NCT04033354	mAb	III	HLX10 in combination with chemotherapy (carboplatin and nab paclitaxel)
NCT03228667	haNK cells in combination with other immunotherapies/PD-1/PD-L1 checkpoint inhibitor	II	N-803 + pembrolizumab + PD-L1 t-haNK N-803 + atezolizumab + PD-L1 t-haNK N-803 + avelumab + PD-L1 t-haNK N-803 + durvalumab + PD-L1 t-haNK

BiKE: bispecific killer cell engager; mAb: monoclonal antibody; NKTR-214: bempegaldesleukin (IL-2 pathway agonist); hank: high affinity natural killer cell; ICI: immune checkpoint inhibitor.

## Data Availability

Not applicable.
